# *Culicoides* Species of the Rain Forest Belt of the Littoral Region of Cameroon: Their Incrimination in the Transmission of *Mansonella perstans*

**DOI:** 10.3390/pathogens13020146

**Published:** 2024-02-06

**Authors:** Rene Ebai, Chi Anizette Kien, Fanny Fri Fombad, Frederick Esofi, Emmanuel Ouam, Anna Ning Ntuh, Glory Ngongeh Amambo, Victor Narcisse Tchamatchoua Gandjui, Valerine Chawa Chunda, Franck Nietcho, Lucy Cho Nchang, Chefor Magha, Jerome Fru Cho, Mathias Eyong Esum, Peter Ivo Enyong, Kenneth Pfarr, Achim Hoerauf, Manuel Ritter, Samuel Wanji

**Affiliations:** 1 Parasite and Vector Research Unit (PAVRU), Department of Microbiology and Parasitology, University of Buea, Buea P.O. Box 63, Cameroonkienchi91.kc@gmail.com (C.A.K.); aning9551@gmail.com (A.N.N.); gvictornarcisse@gmail.com (V.N.T.G.); chundavalerinne@gmail.com (V.C.C.); cheformagha1@gmail.com (C.M.);; 2 Research Foundation for Tropical Diseases and the Environment (REFOTDE), Buea P.O. Box 63, Cameroon; 3 Institute of Medical Microbiology, Immunology and Parasitology (IMMIP), University Hospital Bonn (UKB), 53127 Bonn, Germanyachim.hoerauf@ukbonn.de (A.H.); 4 German Centre for Infection Research (DZIF), Partner Site Bonn-Cologne, 53127 Bonn, Germany; 5 German-West African Centre for Global Health and Pandemic Prevention (G-WAC), Partner Site Bonn, 53127 Bonn, Germany

**Keywords:** *Culicoides* species, relative abundance, midges, biting cycle, *Culicoides milnei*, *Mansonella perstans*, L3 infective larvae, Mp419 LAMP assay

## Abstract

Biting midges belonging to the genus *Culicoides* are tiny stout-shaped hematophagous insects and are thought to transmit the filarial nematode *Mansonella perstans*. Little is known about the *Culicoides* fauna in the rain forest belt of the Littoral Region of Cameroon. This study was designed to investigate the diversity, abundance and distribution of *Culicoides* spp. and their role as the purported vector(s) of *M. perstans*. Overnight light trap collections and human landing catches (HLCs) revealed eight species of *Culicoides* with *C. grahamii* being the most abundant species followed by *C. milnei*. Four anthropophilic species (*C. inornatipennis*, *C. grahamii*, *C. fulvithorax* and *C. milnei*) were determined by the HLCs with a higher abundance in the 4–6 p.m. collections. The drop trap technique and Mp419 LAMP assay confirmed *C. milnei* to be the most efficient vector in enabling the development of the microfilarial stage to the infective larval form of *M. perstans*. The LAMP assay also revealed that natural transmission of this nematode is fostered by *C. milnei* and *C. grahamii* in the wild. In conclusion, *C. milnei* was shown to be the main vector of *M. perstans* in the rain forest belt of the Littoral Region of Cameroon.

## 1. Introduction

Ceratopogonidae form a family of small nematocera midges, usually less than 3–4 mm in length. Worldwide, over 1400 species of these biting midges have been described in the genus *Culicoides*, except in Antarctica and New Zealand [1,2]. The abundance, diversity and seasonal occurrence of *Culicoides* species are largely determined by the availability of moisture-rich habitats that play crucial roles in the growth and survival of the immature stages [2,3]. These midges have a complete life cycle lasting 2–6 weeks; males and females feed on plant juice, while females require a blood meal for egg maturation. Biting is around dawn and dusk, although often at other times, and females lay between 70 and 180 eggs 3–4 days after a blood meal [4,5]. The presence of animals, especially livestock, such as cattle, horses and sheep, play an important role in the abundance, diversity, distribution and biting nuisance of *Culicoides* species. 

*Culicoides* species possess the ability to transmit various filarial parasites of the genus *Mansonella* and viruses (African horse sickness disease virus (AHSV), bluetongue virus (BTV), epizootic hemorrhagic virus (EZHV), Schmallenberg virus (SBV)) to humans and domestic and wild animals [6,7,8,9]. Prevention and control strategies not only include vector control with the use of adulticides and larvicides to target the developmental stages but also the use of insect repellents, wearing of protective clothing, avoiding visiting midge-infested areas and health education to reduce the number of direct biting contacts (DBCs) with these vectors.

Mansonellosis is caused by different *Mansonella* species, but knowledge about this disease is mainly based on *Mansonella perstans*. Mansonellosis is not associated with distinct severe clinical symptoms and is thus not considered as a public health problem, although there are clinical reports that associate infections with symptoms [5,10]. One explanation of the asymptomatic infection outcome is that *M. perstans* downregulate the host immune system [11]. Thus, exact numbers of infected individuals are difficult to assess, but it is estimated that 114 million individuals are infected with *M. perstans* in West, East and Central Africa and in some neo-tropical regions of Central and South America, and another 600 million people live at high risk in 33 African countries [5,10,12]. In 1928, the first definitive association between the parasite and these vectors was postulated, showing that wild *Culicoides grahamii* and *C. austeni* allowed the development of *Acanthocheilonema* (*Mansonella*) *perstans* [13]. Discrepancy arose later as others reported on the uptake of the *Mansonella perstans* microfilaria by *Culicoides inornatipennis* [4,14,15,16]. Recently, we reported on the indisputable role played by *Culicoides milnei* in the transmission of *M. perstans* out of the eight identified species in the south-west region of Cameroon [3]. However, the epidemiology of *Culicoides* and their role in the transmission of *M. perstans* in other regions of Cameroon, such as the Littoral Region, remain unknown. Thus, this study aimed to identify the different *Culicoides* species in the rain forest belt of the Littoral Region of Cameroon and to determine their role in the transmission of *M. perstans*, the most widespread filarial nematode in sub-Saharan Africa [17]. We also aimed to investigate the differences, if any, as a result of human activities that exist between the ecology of this rain forest belt and that of the south-west region. Different collection techniques were employed to evaluate the abundance, diversity and distribution of the *Culicoides* species and to establish the biting density of the supposed vector(s) of this filarial nematode.

## 2. Materials and Methods

### 2.1. Study Sites

This study was carried out in 8 selected communities (Badjoki, Banya II, Bonabekeng, Ndokndak, Nkongmalang, Nyamtan, Salaka and Yangom) in 2 health districts (Yabassi (4°27′16″ N, 9°58′56″ E) and Loum (4°42′58″ N, 9°44′47″ E)) of the Littoral Region of Cameroon (4°16′82″ N, 10°08′07″ E). The Littoral Region occupies the rain forest area and the littoral zone (mangroves biome). An estimated 90% of the population of each community are smallholder farmers, while the others are involved in hunting and fishing activities. There are no industrial livestock farms, but a few families raise brood chickens, pigs or cattle and cultivate food crops mostly around their homes. During the dry season, the daily temperature ranges from 25 to 35 °C and in the rainy season 25 ± 5 °C, with an annual average temperature of 26.8 °C. Rainfall is usually convectional with an average total accumulation estimated at 18.3 inches and an average annual relative humidity of 70 ± 5%.

### 2.2. Study Design

This study was conducted between September 2021 and April 2022 in three parts: the parasitological survey, the entomological survey and the molecular aspect. In the parasitological survey, blood samples were collected from participants to detect the blood-dwelling unsheathed microfilaria (mf) of *M. perstans*, while the entomological survey involved the use of different trapping methods (human landing catches (HLCs) and drop trap (DT) (experimental arm) and overnight UV-light trap (LT) collections) to collect experimentally infected and wild *Culicoides* species. Both the experimental arm and the molecular aspect were used to confirm the transmitting vector(s) of *M. perstans*.

### 2.3. Collection of Adult Culicoides Midges Using CDC Miniature UV-Light Trap Technique

There were alterations in the number of overnight light trap collections as a result of poor road access into some communities. Collections of midges were carried out using 6 V and 12 V CDC miniature black UV-light traps (Model 512, John W. Hock Company, Gainesville, FL, USA). The number of collection days ranged from 1 to 3 nights in some communities, and four UV-light traps were mounted at strategic positions in each community around human homes. The collection time was fixed from 6 p.m. to 6 a.m. each working day and the contents of the collection cups from the light traps were emptied every hour into labeled 50 mL tubes. At the end of the collection period, the samples were randomly separated into two groups and stored in 80% alcohol: one for the entomological arm and the other for the molecular aspect. All samples were then transported to the research laboratory in Manjo.

### 2.4. Collection of Adult Culicoides Midges Using the Human Landing Catch (HLC) Technique

In determining which *Culicoides* species target humans, adult midges were collected using the HLC method from 5 selected communities (Bonabekeng, Nyamtan, Ndokndak, Salaka and Badjoki). Collection took place in the morning (6–9 a.m.) and evening (4–6 p.m.) and this was completed by 4 well-trained collectors (who worked in all five communities) dressed in protective clothing against midges and other hematophagous insect bites. The collectors were positioned in four different areas around some selected homes in each community. The collection was performed for 3 h/morning and 2 h/evening in each day, making a total of 5 collection hours daily. The midges were aspirated as soon as they landed and before taking a blood meal on the midge collectors. The aspirate was blown gently into labeled hourly netted plastic cups and placed in cooler boxes before transportation to the laboratory for speciation and preservation for onward uses. 

### 2.5. Collection and Maintenance of Engorged Culicoides Midges from a Mansonella perstans Positive Volunteer Using the Drop Trap Technology

Collections were completed from 6 p.m. to 6 a.m. on each collection day, and for four nights in some selected communities. In total, 4 donors participated in the study. A *M. perstans* microfilaremic volunteer (donor) agreed to sit under a rectangular netting cage trap (3 × 2 × 2 m). During collection, the netting material of the cage was raised for 15–20 min to allow enough time for contact between the donor and the vectors. The netting material was then lowered for 15–20 min, a period expected for the majority of the attracted midges to be fully engorged. After this time, the blood-fed midges were gently aspirated with the help of flash torches by skilled midge collectors and blown into labeled 50 mL tubes (Corning Inc., Corning, NY, USA) 3/4 filled with plaster of Paris (POP) and covered with a *Culicoides* netting material over a perforated lid. The collected specimens were transported to the research laboratory for maintenance. During maintenance, 15% sucrose solution moistened on cotton pads was placed on top of the netting material on the rims of the tubes for the engorged *Culicoides* species to feed from below. Additionally, 2–3 drops of distilled water were added daily using a 10 mL syringe to keep the bottom of the tubes moist. After 12 days post-infection under laboratory rearing conditions, the specimens for dissection were knocked down in a Tween 20 killing solution for 1–2 min and carefully removed into a second petri-dish containing a rinsing distilled water solution. The knocked-down midges were placed on dissecting slides containing 1 drop of an incomplete culture medium (RPMI-1640 medium; Sigma-Aldrich, Munich, Germany) supplemented with a 2% antibiotic cocktail of penicillin–streptomycin–neomycin (Thermo Fisher Scientific, Schwerte, Germany). The body segments of the specimens were separated into head, thorax and abdomen to allow any larvae to exit the body parts. Infective larvae (L3s) and other sub-stages L1 and L2 were isolated, and the numbers of larvae were recorded on dissection sheets. 

### 2.6. Susceptibility of Culicoides Species to Uptake of Mansonella perstans Microfilaria

In order to attest that *Culicoides* species were susceptible to the uptake of the microfilariae of *M. perstans*, selected engorged *Culicoides* species were dissected in the field after 1 and 2 days post-infection and examined under light microscopes by field entomologists. The number and larval stage(s) were recorded on dissection sheets. 

### 2.7. Morphological Identification of Adult Culicoides Species

Several identification keys were employed for the speciation of *Culicoides* species [18,19,20,21]. Morphological identification was centered on the examination of the wing pigmentation pattern by a microscopist using a dissecting microscope (Leica M80/10450167; Motic, Wetzlar, Germany). In situations of unresolvable evidence on the wing structure, other morphological traits, e.g., maxillary palps or mandibular, the inter-ocular space and male genitalia, were considered.

### 2.8. Colorimetric LAMP Assay to Detect M. perstans in Culicoides Species Collected Using the UV-Light Trap and the HLC Techniques

Wild-caught *Culicoides* biting midges were morphologically identified to species levels and later separated into different pools following the species for DNA to be extracted and pool screening by the Mp419 LAMP assay. LAMP assay targeting the *M. perstans*- specific consensus sequence (Mp419) was carried out as previously described by [22] with slight modifications. The primers used consisted of the following sequence (5′–3′): F3-ACAGTTGATTATTTGAAGGTGCTR, FIP-TGTGAGCACATTTCAGTAAGT-GATGAAATCCACTAAATTCWC, BIP-GGATTCTTTCTAAAAGTTGAG-GATCGATTTCGTTAAAAACAGY, B3-AYAATGATTATTTYTAAAGAATC, LF-AGACTTGATTACTGTTTGG and LB-ACAATTTGGTAATCGCTTAAACTG. Briefly, LAMP reactions contained 1.6 μM of primers, FIP and BIP, 0.2 μM of F3 and B3, 0.4 μM of LF and LB, 10 μL of WarmStart Colorimetric LAMP 2X Master Mix (New England Biolabs Inc., Ipswich, MA, USA) and 2 μL of template DNA, or molecular biology grade H_2_O for non-template controls (NTCs), in a total volume of 20 μL. Reactions were incubated at 63 °C for 40 min in a GeneAmp^®^, PCR System 9700 Thermal Cycler (Applied Biosystems, Waltham, MA, USA). Samples were considered positive for *M. perstans* DNA if an obvious color change from pink to yellow was observed, while negative samples remained pink. Non-template controls were included in each LAMP reaction.

### 2.9. Data Collection and Analysis

Data were collected and compiled on record sheets which were later entered into a template in Microsoft Excel 2010 (Microsoft, Redmond, WA, USA). The data were then exported to IBM SPSS statistics software version 25 for statistical analysis. Prism software (GraphPad Prism 8.0.2 Software, Graph Pad, San Diego, CA, USA) was used for plotting graphs. The diversity and abundance of *Culicoides* species were expressed as the number of different species of *Culicoides* collected per site. The number of midges captured by a trap in a day was expressed as the number of midges collected divided by the number of days divided by the number of traps (midge/day/trap). The number of midges per person per day was expressed as the number of midges attempting to bite a collector divided by the product of the number of collection days and the number of collectors (midge/person/day). A chi-square test was used to compare the abundance of different species across the communities and the proportions of adult species collected by the different trapping techniques. The infection rate was determined as the proportion of infected midges to the total number of midges dissected. The infection and infective rates from pool screening were computed using the algorithm described by Katholi and colleagues [23]. *p*-values less than 0.05 (*p* ≤ 0.05) were considered statistically significant.

## 3. Results

### 3.1. Collection of Culicoides Midges Using CDC Miniature UV-Light Traps

**Figure 1:**
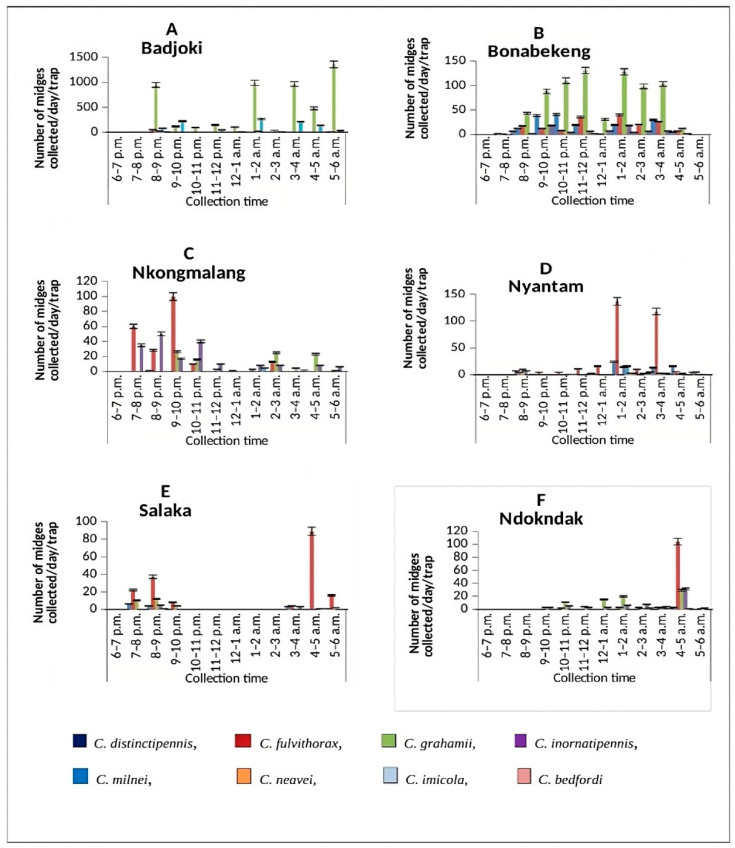
Hourly abundance of *Culicoides* midges collected using the UV-light trap technique in (**A**) Badjoki, (**B**) Bonbekeng, (**C**) Nkongmalag, (**D**) Nyantam, (**E**) Salaka and (**F**) Ndokndak. Bars show standard error of the mean.

A total of 9127 midges were collected from all six selected sites (Badjoki, Bonabekeng, Ndokndak, Nkongmalang, Nyantam and Salaka) between September 2021 and April 2022. *C. grahamii* was the most abundant species (*n* = 6100; 66.8%) and *C. bedfordi* was the least abundant (*n* = 3; 0.03%). The highest number of specimens was collected from Badjoki (*n* = 6501, 71.23%), with the fewest from Salaka (*n* = 229, 2.51%). There was a significant difference in the total number of *Culicoides* midges collected hourly from the different study sites (χ^2^ = 3530.4, df = 88, *p* < 0.0001; Figure 1). An overview of the collected *Culicoides* midges at the different study sites using UV-light traps is presented in Table 1.

### 3.2. Collection of Culicoides Midges Using the Human Landing Catch (HLC) Technique

In total, 2101 midges were collected by the HLC technique, and we found four anthropophilic species (*C. milnei*, *C. inornatipennis*, *C. grahamii* and *C. fulvithorax*). The most abundant species was *C. grahamii* (*n* = 1823; 86.77%) followed by *C. milnei* (*n* = 225, 10.71%), with a higher abundance in the evening (*n* = 224, 99.56%) than in the morning (*n* = 1, 0.04%) collections, while the least abundant species was *C. fulvithorax* (*n* = 3, 0.14%). An overview of the collected *Culicoides* midges using the HLC technique is presented in Table 2. Interestingly, the total number of *Culicoides* midges collected in the morning (*n* = 1377, 65.54%) was higher than those collected in the evening (*n* = 724, 34.46%), but these differences between the morning and evening collections were not statistically significant even at the mean hourly morning (*n* = 23, 56.1%) and mean hourly evening (*n* = 18, 43.9%) collections (χ^2^ = 0.78, df = 1, *p* = 0.38).

### 3.3. Isolation of L3s from Laboratory-Reared Engorged Culicoides Midges

A total of 793 engorged specimens were dissected from five morphological identified *Culicoides* species 12 days post-infection. *M. perstans* larvae were detected in 280 midges (35.31%). A total of 474 infective larvae (L3) were generated from the 280 engorged midges. In general, more L3s were isolated from the head (*n* = 264, 56.70%), followed by the thorax (*n* = 132, 27.85%) and the abdomen (*n* = 78, 16.46%) of the dissected midges. The highest number of infective larvae were recovered from *C. milnei* (*n* = 469, 98.95%), followed by *C. grahamii* (*n* = 4, 0.84%). Out of the four participants (L012, E005, C003 and M013) recruited for the study, the highest number of engorged samples (*n* = 296, 37.33%) were dissected from donor L012, who happened to also have the highest microfilaria load of 16,450 mf/mL of blood; the fewest number of samples (*n* = 77, 9.71%) was dissected from donor C003 (6000 mf/mL) (Table 3). In general, there was a significant difference in the total number of infective larvae generated between donor L012 and donor M013 (*p* < 0.001).

### 3.4. Susceptibility of Culicoides Midges to the Uptake of Mansonella perstans Microfilariae

A total of 32 engorged specimens were morphologically identified as *C. milnei* (*n* = 22) and *C. grahamii*, (*n* = 10) using the drop-trapping technique. Out of the 22 engorged *C. milnei*, 10 were taken from Day 1 and 12 were selected from Day 2 post-infection, while 5 engorged *C. grahamii* were each selected from Days 1 and 2. All 10 (100%) engorged *C. milnei* midges from Day 1 were positive for *M. perstans* microfilariae, while 10 out of the 12 midges (83.33%) from Day 2 were positive for *M. perstans* microfilariae. In contrast, one (*n* = 1, 20%) *C. grahamii* midge each from Days 1 and 2 were positive for *M. perstans* microfilariae (Table 4), confirming that *C. milnei* is the major transmitting vector for mansonellosis [3]. 

### 3.5. Hourly Collection Cycle of Culicoides Midges Using the UV-Light Trap Technique

**Figure 2:**
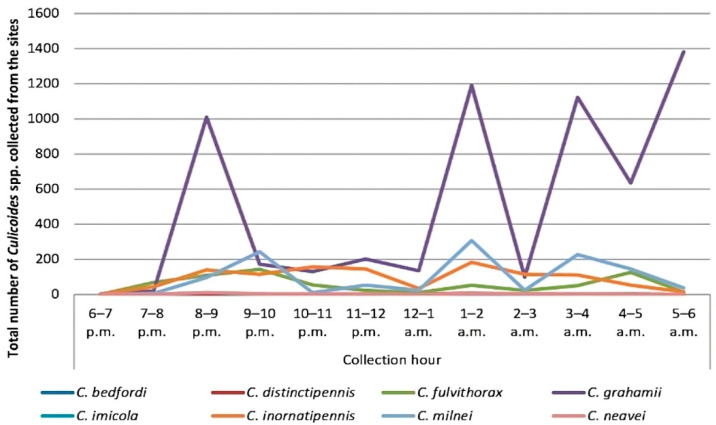
Overall UV-light trap visiting cycle of *Culicoides* midges in the Littoral Region.

**Figure 3:**
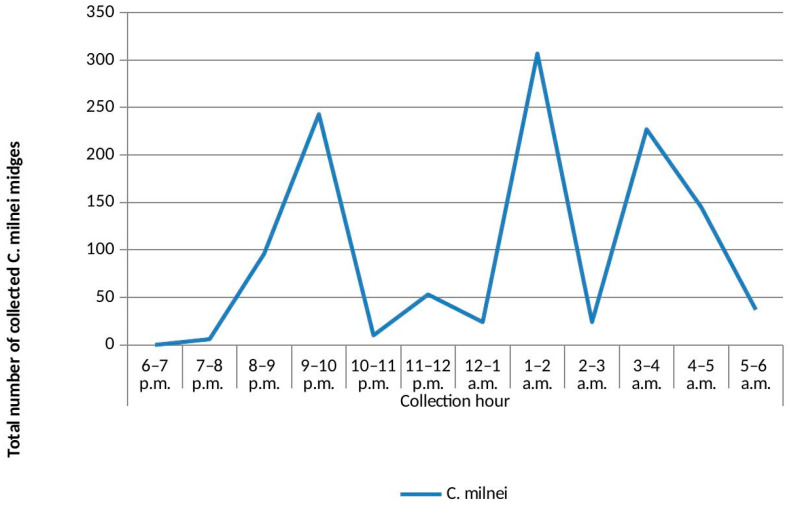
UV-light trap visiting cycle of *Culicoides milnei* in the Littoral Region.

Overall, the highest collection hour was from 1 to 2 a.m. (*n* = 1745, 19.12%), followed by 3–4 a.m. (*n* = 1517, 16.62%), and the least collection hour was 6–7 p.m. (*n* = 4, 0.04%). The highest collection peak from *C. grahamii* was between 5 and 6 a.m. (*n* = 1382, 15.14%), followed by *C. milnei* with three distinct collection peaks: 1–2 a.m. (*n* = 307, 26.19%), 9–10 p.m. (*n* = 243, 20.73%) and 3–4 a.m. (*n* = 227, 19.37%) (Figure 2). However, the least-collected species were *C. bedfordi* and *C. distinctipennis* with collection peaks of 6–7 p.m., 7–8 p.m. and 5–6 a.m. There was a significant difference in the hourly collection of the different species following the peaks (*p* < 0.0001). As *C. milnei* is the major vector of *M. perstans*, the trap visiting cycle of this midge species is separately presented in Figure 3.

### 3.6. Mansonella Perstans Infection in Wild-Caught Culicoides Midges Collected Using the UV-Light Trap and HLC Techniques as Detected by Mp419 LAMP

A total of 460 pools from 15,670 species of different midges collected using the UV-light-trapping method was grouped to species levels. All species were grouped in pools of 50 each, except *C. milnei*, which was grouped in pools of 10 each. *C. grahamii* had the highest number of pools (*n* = 225, 48.9%) followed by *C. milnei* (*n* = 181, 39.3%), and *C. neavei* had only one pool (0.2%). Among the different *Culicoides* species from the UV-light traps in the different pools, we observed two pools each from *C. milnei* and *C. grahamii* that were positive by the LAMP assay, with infection rates of 0.2% for *C. milnei* and 0.04% for *C. grahamii* as indicated with a visible color change from pink to yellow. The LAMP assay was also performed on DNA extracted from 150 wild-caught *C. grahamii* using the HLC technique and grouped into three pools of 50 midges/pool. Out of the 150 *C. grahamii* samples analyzed, none were positive with the infection by the LAMP assay. 

## 4. Discussion

In areas where *Culicoides* are abundant, *Culicoides* are a biting nuisance to humans and domestic and wild animals [1,2,24]. Overall species diversity was detected to be higher with the UV-light trap technique (*Culicoides grahamii*, *C. milnei*, *C. fulvithorax*, *C. neavei*, *C. inornatipennis*, *C. bedfordi*, *C. imicola* and *C. distinctipennis*) compared to the HLC technique with four different *Culicoides* species. This shows that collections with the UV-light-trapping technique were more elaborate in determining *Culicoides* species diversity, as confirmed with previous studies from Ghana [25] and the south-west region of Cameroon [3] where species diversity was also higher with UV-light trap techniques, having seven and eight species compared to the HLC technique with one and four *Culicoides* species, respectively. *C. kumbaensis* from the south-west region was replaced by *C. distinctipennis* in this study, although both species have previously been reported in former British Southern Cameroon [4,13]. These differences in species diversity and abundance may be influenced by the type of ecosystem, ecological alterations, periods of collection or the proximity of other animals [26,27]. For instance, the presence of *C. distinctipennis* in the rain forest area of the Littoral Region may be accounted for by the presence of boggy ground that is thinly covered with grass and decumbent plants [4]. The overall UV-light trap visiting cycle of *Culicoides* species in the Littoral Region reveals the peak activity hours of the different *Culicoides* species. When replacing the light traps with humans, then the peak active hours become the peak biting hours for the *Culicoides* midges. We extracted the light trap visiting cycle only for *C. milnei* to demonstrate the ideal peak transmission potential hours of this *M. perstans* vector in such communities. This information is very important when drawing up implementation research programs of vector control strategies against this species and other biting midges.

With HLC, we identified four anthropophilic species: *C. inornatipennis*, *C. grahamii*, *C. fulvithorax* and *C. milnei*, with *C. grahamii* being the most abundant species (86.77%). Exceptionally, in the UV-light trap collection, *C. grahamii* was again the most abundant (66.8%) followed by *C. milnei* (12.8%). The high abundance and consequently the biting nuisance of *C. grahamii* and *C. milnei* from the UV-light traps, HLC and other methods of collection indicate that these anthropophilic *Culicoides* species possess the same ecological niche, supposing that the year-round cultivation of banana and plantain, especially around human dwellings, greatly supports the survival and development of their immature stages in this region than the other *Culicoides* species [4,28]. Others also captured these four species as anthropophilic species, although some of the studies did not capture all four species at the same time [3,4,20,29,30].

In the hourly collection of the light trap visiting cycle, the highest collection peak was observed with *C. grahamii* at 5–6 a.m., while *C. milnei* was characterized by three peaks, with the highest in the early morning at 1–2 a.m., followed by 9–10 p.m. and lastly at 3–4 a.m. In contrast, *C. grahamii* had the highest collection peak at 3–4 a.m., while *C. milnei* was characterized by two active peaks at 10–11 p.m. and 2–4 a.m. in the south-west region [3]. This paradigm may be the result of the influence of abiotic factors like temperature and annual precipitation rate in both regions.

With the drop trap technique, we identified five *Culicoides* species (*C. milnei*, *C. inornatipennis*, *C. grahamii*, *C. fulvithorax* and *C. neavei*) and all five species had been previously reported in the south-west of Cameroon using the same method of collection [3,4]. *C. milnei* was the most abundant species collected using this technique (56.12%), followed by *C. grahamii* (23.20%). This may sound contradictory as *C. grahamii* was the most abundant species in the ‘overnight’ UV-light trap collections, but it was not the most abundant in the overnight drop trap collection technique in both regions. The reason might be that *C. milnei* is more active in complete darkness (absence of light source), whereas *C. grahamii* is only active in the presence of a light source. The fact that *C. grahamii* was the most abundant species in the overnight light trap collection does not make it a nocturnal species, as the species was only attracted to its bait (light). In contrast, *C. milnei* was the most abundant species in the overnight drop trap collection because of the optimal environmental conditions, i.e., darkness. However, both species are attracted by body odor or pheromones to their vertebrate hosts. 

Upon dissection, 474 L3s were recovered, and 98.95% of the L3 were isolated from *C. milnei*, while 0.84% came from *C. grahamii*. In this arm, *C. milnei* proved to be the more competent species in enabling the development of *M. perstans* sausage stages than *C. grahamii*, confirming previous studies that have stated that *C. grahamii* is a very poor vector of *M. perstans* [4,15,29]. Based on these findings, we postulate that *C. grahamii* is an inefficient vector of *M. perstans*, while *C. milnei* is the most competent vector of *M. perstans* and a principal night biter [3,4,31].

For over 30 years now, molecular techniques such as PCR have been used in research laboratories as confirmatory tests; however, the training of personnel and the relatively expensive equipment required make them unsuitable for field use. The advent of isothermal amplification methods is intended, particularly, for low-resource settings [32]. Loop-mediated isothermal amplification (LAMP) is now the most widely adopted of these molecular methods [33]. In this study, the Mp419 LAMP assay was used to identify *M. perstans* in wild-caught *Culicoides* species from different sites using the UV-light trap and HLC techniques. Overall, only *C. milnei* and *C. grahamii* samples had positive visual signals when the Mp419 LAMP assay was used on samples collected with the UV-light traps, suggesting that these two species are the vectors in the natural transmission of *M. perstans*. In contrast, *C. grahamii* samples collected with the HLC technique were all negative for the *M. perstans* parasite. This may be due to the midges not taking an infected-blood meal before being captured or the lower prevalence of the infection in these sites. The UV-light trap collection may be a better method for midge collection than HLC when investigating the prevalence of the infection either by microcopy or molecular assay. Indeed, LAMP was recently used to detect *M. perstans*, *Loa loa* and *Onchocerca volvulus* infections in both engorged and wild-caught *Culicoides*, *Chrysops* and *Simulium* flies, respectively [34,35,36].

In this study, in nature, *C. grahamii* were more numerous than *C. milnei*; however, experimentally, *C. milnei* were superior to *C. grahamii* for *M. perstans* larval development and the major vector of *M. perstans*. This is consistent with earlier studies, which identified *C. grahamii* to be a potential vector of *M. streptocerca* rather than of *M. perstans* [37].

## 5. Conclusions

In summary, this study mirrors observations made four years ago in the south-west region of Cameroon; eight *Culicoides* species are present in the Littoral Region of Cameroon. The UV-light trap visiting cycle revealed *C. milnei* to have three collection peaks: the highest at 1–2 a.m., followed by 9–10 p.m. and lastly 3–4 a.m. The results showed that the ecological niche of the *Culicoides* species in the Littoral Region’s rainforest area does not differ much from that in the south-west region of Cameroon. Meanwhile the experimental arm and the LAMP assay technologies identified *C. milnei* to be the most competent vector of *M. perstans* with *C. grahamii* and *C. milnei* being responsible for the natural transmission of *M. perstans*.

## Figures and Tables

**Table 1:** *Culicoides* midges collected using UV-light traps at 6 study sites.

Species	Sites						Total (%) from Night Traps *n* = 22	Midge/Trap/Night
	Bonabekeng *n* = 4	Nkongmalang *n* = 2	Badjoki *n* = 4	Salaka *n* = 4	Nyantam *n* = 4	Ndokndak *n* = 4		
*C. milnei*	76 (19)	7 (3.5)	1054 (263.5)	09 (2.3)	22 (5.5)	4 (1)	1172 (12.8)	53.3
*C. grahamii*	168 (42)	98 (49)	5257 (1314.3)	176 (44)	315 (78.8)	86 (21.5)	6100 (66.8)	277.3
*C. inornatipennis*	744 (189)	182 (91)	67 (16.8)	28 (7)	28 (7.0)	64 (16)	1113 (12.2)	50.6
*C. fulvithorax*	173 (43.3)	215 (107.5)	87 (21.8)	14 (3.5)	69 (17.3)	115 (28.8)	673 (7.4)	30.6
*C. distinctipennis*	0 (0)	1 (0.5)	12 (3)	0 (0)	0 (0)	0 (0)	13 (0.1)	0.6
*C. neavei*	0 (0)	0 (0)	24 (6)	2 (0.5)	13 (3.3)	0 (0)	39 (0.4)	1.8
*C. imicola*	0 (0)	0 (0)	0 (0)	0 (0)	14 (3.5)	0 (0)	14 (0.2)	0.6
*C. bedfordi*	0 (0)	0 (0)	0 (0)	0 (0)	0 (0)	3 (0.75)	3 (0.03)	0.1
Total	1161	503	6501	229	461	272	9127 (100)	414.9

(Numbers in parentheses represent the number of *Culicoides* per trap per night (midge/trap/night) for each species in the various sites; *n* = number of traps).

**Table 2:** *Culicoides* midges collected using the human landing catch (HLC) technique.

Species	Sites										Total		All Total (%)	C/M/H	
	Badjoki		Bonabekeng		Ndokndak		Nyantam		Salaka						
	M	E	M	E	M	E	M	E	M	E	M	E		M	E
*C. grahamii*	1039	381	199	76	60	4	15	10	32	7	1345	478	1823 (86.77%)	1.12	0.60
*C. milnei*	1	103	0	15	0	4	0	20	0	82	1	224	225 (10.71%)	0.00	0.28
*C. fulvithorax*	0	0	0	0	2	0	0	1	0	0	2	1	3 (0.14%)	0.00	0.00
*C. inornatipennis*	13	0	5	10	1	2	6	4	4	5	29	21	50 (2.38%)	0.02	0.03
Total	1053	484	204	101	63	10	21	35	36	94	1377	724	2101	1.14	0.91
	1537 (73.16%)		305 (14.52%)		73 (3.47%)		56 (2.67%)		130 (6.19%)		2101			1.03	

(Number of collectors—4; number of working hours (morning)—3; number of working hours (evening)—2; number of collection days—4; total number of morning working hours—12; total number of evening working hours—8.) M—morning; E—evening; C/M/H—number of *Culicoides* per man per hour.

**Table 3:** Recovered *Mansonella perstans* larvae from *Culicoides* midges dissected 12 days post-infection (E005, M013, C003 and L012 represent the specific donor code; mf/mL—number of microfilarial per milliliter peripheral blood).

Donor (mf/mL)	Species	Number of Dissected Midges	Number of Infected Midges	Number of Isolated Larvae										Infection Rate
				Total Number of Recovered L3										
				Total	Head			Thorax			Abdomen			
					L1	L2	L3	L1	L2	L3	L1	L2	L3	
**E005** (8000)	*C. milnei*	126	19	20	0	2	17	0	2	1	0	0	2	0.15
	*C. grahamii*	39	00	0	0	0	0	0	0	0	0	0	0	0
	*C. inornatipennis*	19	00	0	0	0	0	0	0	0	0	0	0	0
	*C. fulvithorax*	5	02	0	0	0	0	0	0	0	0	2	0	0.4
	*C. neavei*	1	00	0	0	0	0	0	0	0	0	0	0	0
	Total	190	21	20	0	2	17	0	2	1	0	2	2	0.11
**M013** (750)	*C. milnei*	4	4	25	0	0	9	0	0	10	0	0	6	1.0
	*C. grahamii*	109	2	4	0	0	2	0	0	1	0	0	1	0.02
	*C. inornatipennis*	75	5	0	0	0	0	0	0	0	2	2	0	0.07
	*C. fulvithorax*	42	2	0	0	0	0	0	0	0	0	2	0	0.05
	Total	230	13	29	0	0	11	0	0	11	2	4	7	0.06
**C003** (6000)	*C. milnei*	53	23	47	0	0	33	0	13	10	0	20	4	0.43
	*C. grahamii*	5	0	0	0	0	0	0	0	0	0	0	0	0
	*C. inornatipennis*	10	2	1	0	2	0	0	1	1	0	0	0	0.2
	*C. fulvithorax*	9	1	0	0	0	0	0	0	0	0	1	0	0.1
	Total	77	26	48	0	2	33	0	14	11	0	21	4	0.34
**L012** (16,540)	*C. milnei*	262	206	377	0	8	203	0	9	109	0	4	65	0.79
	*C. grahamii*	31	14	0	0	0	0	0	0	0	10	4	0	0.45
	*C. inornatipennis*	3	0	0	0	0	0	0	0	0	0	0	0	0
	*C. fulvithorax*	0	0	0	0	0	0	0	0	0	0	0	0	0
	Total	296	220	377	0	8	203	0	9	109	10	8	65	0.74
Grand Total		793	280	474	0	12	264	0	25	132	14	35	78	0.35

**Table 4:** Susceptibility of engorged midges to *Mansonella perstans* microfilariae.

Days Post-Infection	Species	Number of Collected Midges	Number of Dissected Midges	Number of Positive Midges (%)	Number of Negative Midges (%)
1	*C. milnei*	10	10	10 (100)	0 (0)
	*C. grahamii*	5	5	1 (20)	4 (80)
2	*C. milnei*	12	12	10 (83.33)	2 (16.66)
	*C. grahamii*	5	5	1 (20)	4 (80)
Total		32	32	22 (68.75)	10 (31.25)

## Data Availability

The data presented in this study are available on request from the corresponding author.
